# External phantom-based validation of a deep-learning network trained for upscaling of digital low count PET data

**DOI:** 10.1186/s40658-025-00745-4

**Published:** 2025-04-16

**Authors:** Anja Braune, René Hosch, David Kersting, Juliane Müller, Frank Hofheinz, Ken Herrmann, Felix Nensa, Jörg Kotzerke, Robert Seifert

**Affiliations:** 1 Department of Nuclear Medicine, University Hospital Carl Gustav Carus at the Technische Universität Dresden, Fetscherstraße 74, 01307 Dresden, Germany; 2 Department of Positron-Emission-Tomography, Helmholtz-Zentrum Dresden-Rossendorf e.V., Institute of Radiopharmaceutical Cancer Research, Bautzner Landstr. 400, 01328 Dresden, Germany; 3 Department of Nuclear Medicine, University Hospital Essen, Hufelandstraße 55, 45147 Essen, Germany; 4 Department of Nuclear Medicine, Inselspital, Bern University Hospital, University of Bern, Freiburgstrasse 18, 3010 Bern, Switzerland; 5 Institute for Artificial Intelligence in Medicine (IKIM), University Hospital Essen, Girardetstraße 2, 45131 Essen, Germany; 6 Institute of Diagnostic and Interventional Radiology and Neuroradiology, University Hospital Essen, Hufelandstraße 55, 45147 Essen, Germany; 7 Carl Gustav Carus Faculty of Medicine, Technische Universität Dresden, Fetscherstraße 74, 01307 Dresden, Germany

**Keywords:** Deep-learning, Denoising, PET image quality, Phantom-based validation

## Abstract

**Background:**

A reduction of dose and/or acquisition duration of PET examinations is desirable in terms of radiation protection, patient comfort and throughput, but leads to decreased image quality due to poorer image statistics. Recently, different deep-learning based methods have been proposed to improve image quality of low-count PET images. For example, one such approach allows the generation of AI-enhanced PET images (AI-PET) based on ultra-low count PET/CT scans. The performance of this algorithm has so far only been clinically evaluated on patient data featuring limited scan statistics and unknown actual activity concentration. Therefore, this study investigates the performance of this deep-learning algorithm using PET measurements of a phantom resembling different lesion sizes and count statistics (from ultra-low to high) to understand the capabilities and limitations of AI-based post processing for improved image quality in ultra-low count PET imaging.

**Methods:**

A previously trained pix2pixHD Generative Adversarial Network was evaluated. To this end, a NEMA PET body phantom filled with two sphere-to-background activity concentration ratios (4:1 and 10:1) and two attenuation scenarios to investigate the effects of obese patients was scanned in list mode. Images were reconstructed with 13 different acquisition durations ranging from 5 s up to 900 s. Image noise, recovery coefficients, SUV-differences, image quality measurement metrics such as the Structural Similarity Index Metric, and the contrast-to-noise-ratio were assessed. In addition, the benefits of the deep-learning network over Gaussian smoothing were investigated.

**Results:**

The presented AI-algorithm is very well suitable for denoising ultra-low count PET images and for restoring structural information, but increases image noise in ultra-high count PET scans. The generated AI-PET scans strongly underestimate SUV especially in small lesions with a diameter ≤ 17 mm, while quantitative measures of large lesions ≥ 37 mm in diameter were accurately recovered. In ultra-low count or low contrast images, the AI algorithm might not be able to recognize small lesions ≤ 13 mm in diameter. In comparison to standardized image post-processing using a Gaussian filter, the deep-learning network is better suited to improve image quality, but at the same time degrades SUV accuracy to a greater extent than post-filtering and quantitative SUV accuracy varies for different lesion sizes.

**Conclusions:**

Phantom-based validation of AI-based algorithms allows for a detailed assessment of the performance, limitations, and generalizability of deep-learning based algorithms for PET image enhancement. Here it was confirmed that the AI-based approach performs very well in denoising ultra-low count PET images and outperforms traditional Gaussian post-filtering. However, there are strong limitations in terms of quantitative accuracy and detectability of small lesions.

**Supplementary Information:**

The online version contains supplementary material available at 10.1186/s40658-025-00745-4.

## Introduction

Artificial intelligence (AI) algorithms are gaining increasing attention in nuclear medicine imaging, especially in the process of PET image reconstruction. In particular, the use of AI-algorithms has already proven to be a particularly promising tool for improvement of image quality of PET scans with reduced acquisition time or dose, as low count images have otherwise poor quality. For example, it has been shown that reducing count statistics to 33% compared to standard acquisition time or dose in routine clinical practice can lead to loss of detectability and image quality, which severely restricts interpretation of the scans [1]. The integration of deep-learning neural network algorithms can help to enhance image quality and especially reduce image noise [2, 3], such that image quality similar to PET scans acquired according to standards in clinical routine (2–3 min per bed position) can be restored in PET scans acquired with reduced scan time or dose. This can be of particular importance to reduce motion artefacts and improve patient comfort, or when examining children who might otherwise have to be sedated in order to remain calm during typical whole-body PET examination times of about 15 min [4]. There is even a commercially available and FDA and CE approved deep-learning-based software solution, which enables a quantitative recovery of SUV values of lesions in PET scans acquired at 50% of the count statistics and restores noise levels equivalent to full PET acquisitions based on EU recommended PET procedure standards (SubtlePET, Subtle Medical, Menlo Park, CA, USA) [1].

Recently, another deep-learning-based algorithm was introduced that generates synthetic full-dose PET image data based on even shorter, ultra-low count FDG PET/CT scans [5]. Briefly, 387 patients were examined in clinical routine using a digital, standard axial field of view PET/CT scanner. Each patient received an ultra-short FDG PET/CT (ExtremePET) scan with scan time durations of about 30 s as well as immediately thereafter a normal acquisition time PET (FullTime-PET) scan with acquisition times of 15 min to 20 min for about 1 m scan length. A Generative Adversarial Network (GAN) was then trained using a pix2pixHD deep-learning network architecture to improve the visual impression and image quality of the ExtremePET scans. The model was specifically trained to denoise ExtremePET data and to generate AI-PET images that feature good detectability of lesions and only small quantitative differences compared to FullTime-PET image data [5]. The pix2pix GAN model was selected because it is designed to generate high-resolution images while preserving details. The different resolution levels used for image generation and discrimination are beneficial for preserving both overall image structure and small details. In particular, denoising of quickly acquired PET images requires both attention at the global level (e.g. for anatomical structures) for overall noise reduction and attention to details to preserve the detectability of small lesions in particular.

The algorithm has already been validated using FDG PET/CT scans of patients examined in clinical routine. Comparisons of SUV_mean_ and SUV_max_ values of lesions between FullTime-PET and AI-PET scans of 33 patients including 298 lesions showed mean absolute differences of 0.91 ± 1.54 and 1.5 ± 2.46, respectively. A matched-pair comparison of the patient-related detection rate of all lesions was 79%. Non-detected lesions in AI-PET scans had a low volume (1.0 ml) and lower tracer uptake (median SUV_mean_ of 2.7) [5].

In view of the validation based on clinical data, it remains to be clarified whether the underestimation of SUV values in AI-PET scans and the worse detectability especially of small lesions are systematic errors. In addition, the optimal reduction of PET scan duration, which might result in a better detectability but still to an accelerated scanning time, is unknown. Therefore, a systematic phantom-based evaluation of the algorithm is mandatory to assess its capabilities and guide new approaches for AI-improved PET image quality.

Although a phantom does not ideally replicate patient-specific characteristics, validation of an AI algorithm using fillable phantoms offers significant added value compared to a validation on clinical data alone. Standardized phantom-based validations allow for the objective assessment of the performance of an AI algorithm and provide additional information about its strengths and weaknesses. Compared to clinical PET scans, in PET measurements of phantoms the actual size of lesions (in a phantom: spheres) and activity concentrations are well known. This enables precise validation of quantitative accuracy and spatial resolution. In addition, with a phantom, several PET/CT scans of the same object can be acquired under different conditions without having to worry about radiation protection. This makes it possible to analyze imaging properties depending on different acquisition conditions such as sphere-background contrast, attenuation values of the patient (fat content), or the acquisition duration. For example, a stepwise reduction of PET scan statistics allows a systematic evaluation of an AI algorithm and the determination of the limits of how much dose/time reduction can be achieved while maintaining diagnostic image quality. In addition, image noise and thus the noise reduction factors as a function of the input noise can only be reliably measured on phantoms. Assuming a homogeneously distributed radioactivity in each phantom compartment, detection of reduced image noise in the background compartment of a phantom exclusively represents improved image quality. In comparison, in PET scans of patients it is unclear whether image noise in the background or in reference organs such as the liver is caused by limited image statistics or of physiological origin. In addition, phantoms show much more clearly potential artifacts that could be caused by the network architecture. They also make it possible to assess the limits of the network’s applicability. If the AI network works well in a phantom recorded with different scanners and/or reconstructed with different settings, this would indicate a good generalizability of the network.

Therefore, the aim of this study was to qualitatively and semi-quantitatively validate the performance of the deep-learning network presented in [5] using PET-listmode measurements of a NEMA PET body phantom imaged under different acquisition conditions (sphere-background contrast, degree of attenuation) and with varying acquisition durations and scan statistics from ultra-low to high. In addition, the performance of the denoising deep-learning network was compared with conventional Gaussian filtering during the image reconstruction process as a simple and standardized noise reduction approach.

## Methods

To evaluate the performance of the deep-learning neural network presented in [5], a NEMA PET body phantom was filled with [^18^F]FDG with an approximate activity concentration of 5.3 kBq/ml in the background compartment and a sphere-to-background activity concentration ratio (SBR) of about 4:1 (SBR4) and 10:1 (SBR10), respectively. After the PET/CT scan of the NEMA PET body phantom with SBR4 or SBR10 in normal setup (hereinafter referred to as thin phantom setup), the phantom was scanned again after wrapping it in gel cooling packs 1 cm thick containing propylene glycol to simulate attenuation and scatter conditions comparable with those in an obese patient (hereinafter referred to as obese phantom setup). Details about the exact actual activity concentrations at the timepoint of imaging were specified in a previously presented study [6]. At the starting timepoint of imaging, the amount of [^18^F]FDG in the phantoms/FoV were 43.2 MBq (SBR4, thin), 36.92 MBq (SBR4, obese), 52.62 MBq (SBR10, thin), and 44.12 MBq (SBR10, obese).

PET/CT scans of the NEMA PET body phantom were acquired using a digital SiPM-based Biograph Vision 600 PET/CT scanner at the University Hospital Carl Gustav Carus Dresden (Siemens Healthineers, Erlangen, Germany), hereafter referred to as Input-PET.

### FDG PET/CT imaging protocol

Following a low-dose CT used for attenuation correction of the subsequent PET scan, each of the four PET scans (SBR4 or SBR10, thin or obese phantom setup) were acquired in list mode for 900 s using a single bed position and covering an axial field of view (FoV) of 26 cm [7].

Each of the four PET scans (SBR4, SBR10, thin and obese phantom setup) were reconstructed using the entire 900 s dataset as well as shorter time frames: 5 s, 10 s and 20 s to 200 s in 20 s increments to simulate 13 different acquisition durations per PET scan. PET data were reconstructed using three-dimensional ordinary Poisson ordered-subsets expectation maximization with 6 iterations and 5 subsets (6i5s), applying point spread function reconstruction and time of flight measurements (TrueX algorithm) with an image matrix size of 440 × 440, resulting in a voxel size of (1.65 × 1.65 × 1.5) mm^3^. No postfiltering was applied (all-pass filter). Reconstructions were performed with attenuation correction and relative scatter correction.

The Input-PET dataset of the NEMA PET body phantom with 10 s acquisition times in SBR4 and thin phantom setup without any filtering (Input-PET_SBR4, thin, Allpass_) was additionally reconstructed using a Gaussian filter with a filter size of 2 mm full width at half maximum (FWHM, G2, Input-PET_10s,G2_), 5 mm (G5, Input-PET_10s,G5_), 10 mm (G10, Input-PET_10s,G10_), 15 mm (G15, Input-PET_10s,G15_), and 20 mm (G20, Input-PET_10s,G20_). Post-Filter Input-PET scans were qualitatively and semi-quantitatively compared with the AI-PET_SBR4, thin, Allpass_ corresponding to the unfiltered Input-PET scan. The Input-PET_SBR4, thin_ setup was chosen for this analysis since this phantom condition was shown to mimic clinically realistic conditions in FDG PET/CT lymphoma patients [8] and since the lower SBR4 contrast ratio is the more difficult phantom setup to depict. The Input-PET scan with an acquisition duration of 10 s was chosen, because the algorithm was trained to improve PET scans of such short recordings and these represent the most challenging data.

### Evaluation methods

Image statistics of the 52 PET datasets of the NEMA PET body phantom (13 different acquisition durations, SBR4, SBR10, thin and obese phantom setups; hereafter referred to as Input-PET) were compared according to the detected number of true events.

The 52 Input-PET datasets were used as input for the deep-learning neural network presented in [5]. For each of the 52 Input-PETs, the deep-learning neural network generated a new PET dataset, hereafter referred to as AI-PET. The corresponding Input-PET and AI-PET scans were compared qualitatively and semi-quantitatively.

#### Ground truth definition

For a comparison of the AI-PET data with the ground truth, a ground truth dataset was defined for each of the four PET acquisitions that corresponded to the clinical standard in [5] and thus to the image quality of the training datasets of the AI algorithm. The ground truth datasets were determined based on CoV_BG_ as the AI algorithm was trained to improve the visual image quality of low-count PET images and since image noise is a key parameter to characterize image quality. According to Fig. 3 and supplementary Table S.4, the AI algorithm considered a CoV_BG_ of ~ 12% in the SBR4, thin dataset and a CoV_BG_ of ~ 16% in the SBR10, thin dataset as optimal and accordingly tried to achieve a noise level in this range in all AI-PET scans. In conjunction with the image statistics (supplementary Table S.3), this indicated that the AI algorithm was trained on data with a true count rate of ~ 35Mio counts. In conjunction with image statistics shown in the supplementary Table S.3, the following scans were considered as respective ground truth reference: SBR4, thin: 180 s acquisition duration; SBR4: obese: 200 s acquisition duration; SBR10: thin: 140 s acquisition duration; SBR10: obese: 200 s acquisition duration.

#### Image quality: semi-quantitative evaluation

Image quality of Input-PET and AI-PET datasets were compared semi-quantitatively using the software Rover (version 3.0.74 h, ABX, Radeberg, Germany). Three uniform background volumes of interest (VOIs) of 131 ml volume were delimited in each scan. Segmentation of each of the six spheres of the NEMA PET body phantom (diameter: 10 mm, 13 mm, 17 mm, 22 mm, 28 mm, 37 mm) was performed for each of the four PET acquisitions (specific contrast ratio, thin and obese phantom setup) as presented in [6]. Briefly, in analogy to [9], a 3D isocontour at 50% of the maximum pixel value was used for the segmentation of each sphere, considering the activity concentration in the background of the phantom. The same VOIs were used for the analysis of the different frame duration reconstructions of the same phantom setup scan, ensuring that the same VOI was analyzed in each reconstruction.

Image noise (CoV_BG_) was calculated as the coefficient of variation of each of the three background compartments. The mean CoV_BG_ values of the three background VOIs were compared between Input-PET and AI-PET scans.

The contrast-to-noise-ratio (CNR) served as a semi-quantitative measure for lesion detectability and was calculated according to [8] as the difference of the VOI mean of each sphere and the background divided by the mean standard deviation of the activity concentrations of the three background VOIs. As of the Rose criterion which is commonly used to characterize the visibility of objects in PET imaging, spheres with CNR ≥ 5 were considered as visible [10].

#### Image-to-image comparison using image quality measurement metrics (SSIM, PSNR, MAE)

For a direct comparison of each AI-PET with the respective ground truth scan and in accordance with the clinical evaluation in [5], the accuracy of the predicted AI-PET scan compared to the respective unbiased ground truth Input-PET scan was evaluated using the following quantitative image quality measurement metrics: Structural Similarity Index Metric (SSIM), Peak Signal-to-Noise Ratio (PSNR) analyzing SUV_peak_ of the largest sphere (37 mm diameter), and SUV-based Mean Absolute Error (MAE). The metrics were calculated using the software R, version 4.4.2 [11] and using a mask of the entire phantom, excluding the voxel space outside the phantom.

#### Quantitative accuracy: recovery coefficients and SUV

For each phantom sphere, the mean and maximum Recovery Coefficients (RC_mean_ and RC_max_) were determined as the ratio of the measured mean and maximum standardized uptake value (SUV_mean_ and SUV_max_) of the VOI and the actual activity concentration in the phantom sphere at the timepoint of imaging, which was determined using a gamma counter. Differences in SUV_mean_ and SUV_max_ between each AI-PET or Input-PET scan and the respective ground truth Input-PET scan were determined as mean and standard deviation of all voxels within the sphere masks.

## Results

### Statistics

**Fig. 1:**
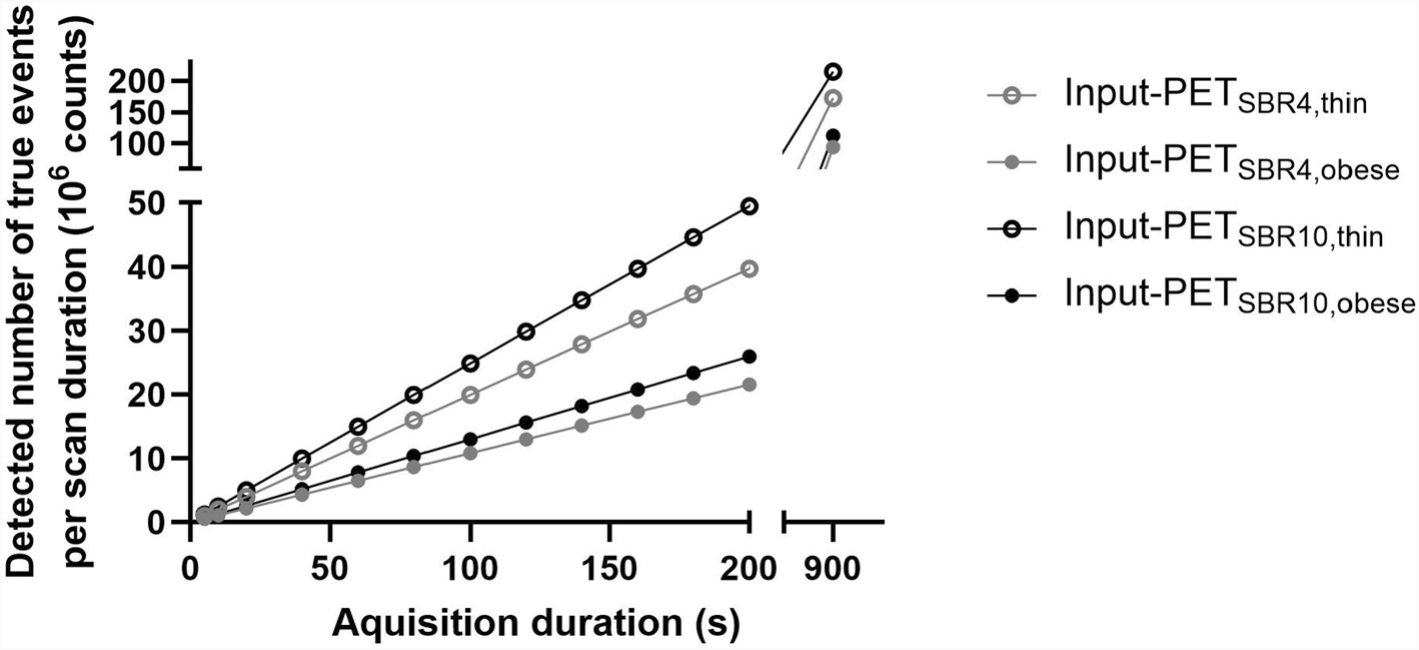
Image statistics. Image statistics were determined as the detected number of true events as a function of acquisition duration (5 s, 10 s, 20 s to 200 s in steps of 20 s and 900 s) of the Input-PET scan with SBR4 (gray) and SBR10 (black) and thin (open circle) as well as obese phantom setups (closed circle). The detected number of true events increased linearly with increasing acquisition duration and was only 54% and 52% in the Input-PET_SBR 4, obese_ and Input-PET_SBR 10,obese_ scans, receptively, relative to the corresponding thin phantom setup scans

Image statics are shown in Fig. 1*.* The detected number of true events per reconstructed Input-PET scan increased linearly with increasing acquisition duration. In Input-PET_SBR4, obese_ and Input-PET_SBR10, obese_ scans, respectively, image statistics were reduced to 54% and 52% relative to the corresponding scans of the thin phantom setup. For thin and obese phantom setup scans, the number of true events detected in SBR10 compared to SBR4 scans were 25% and 20% higher, respectively, due to the varying amounts of activity in the phantom at the timepoint of imaging.

### ***Image quality: qualitative and semi-quantitative evaluation (CoV***_***BG***_*** and CNR)***

**Fig. 2:**
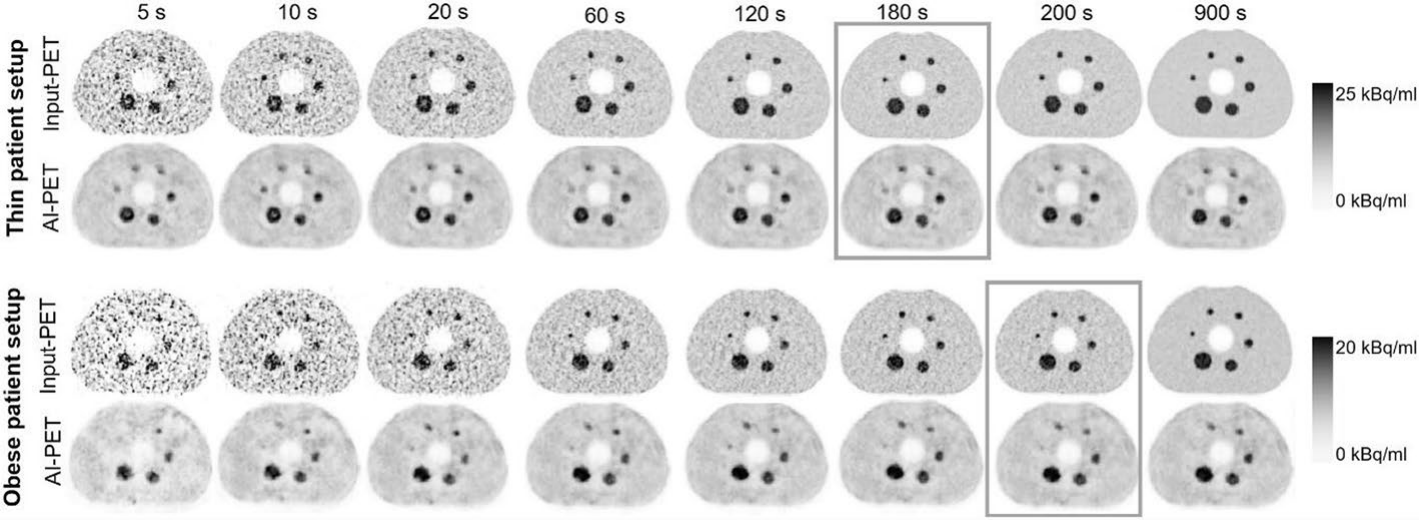
Qualitative comparison of Image Quality between Input-PET, ground truth and AI-PET scans. PET scans of the NEMA PET body phantom with SBR4 as well as the corresponding AI-PET scans are shown at the same axial position for thin and obese phantom setup scans and for different acquisition durations ranging from 5 s (column 1) to 900 s (column 6). For both phantom setups, the Input-PET scan, which was considered as reference ground truth scan, is edged in gray

For qualitative comparison of image quality between low- and high-count Input-PET, corresponding AI-enhanced AI-PET and corresponding ground truth scans, Fig. 2 exemplarily illustrates the Input-PET and AI-PET scans of the NEMA PET body phantom with SBR4 and thin as well as obese phantom setup for different acquisition durations.

**Fig. 3:**
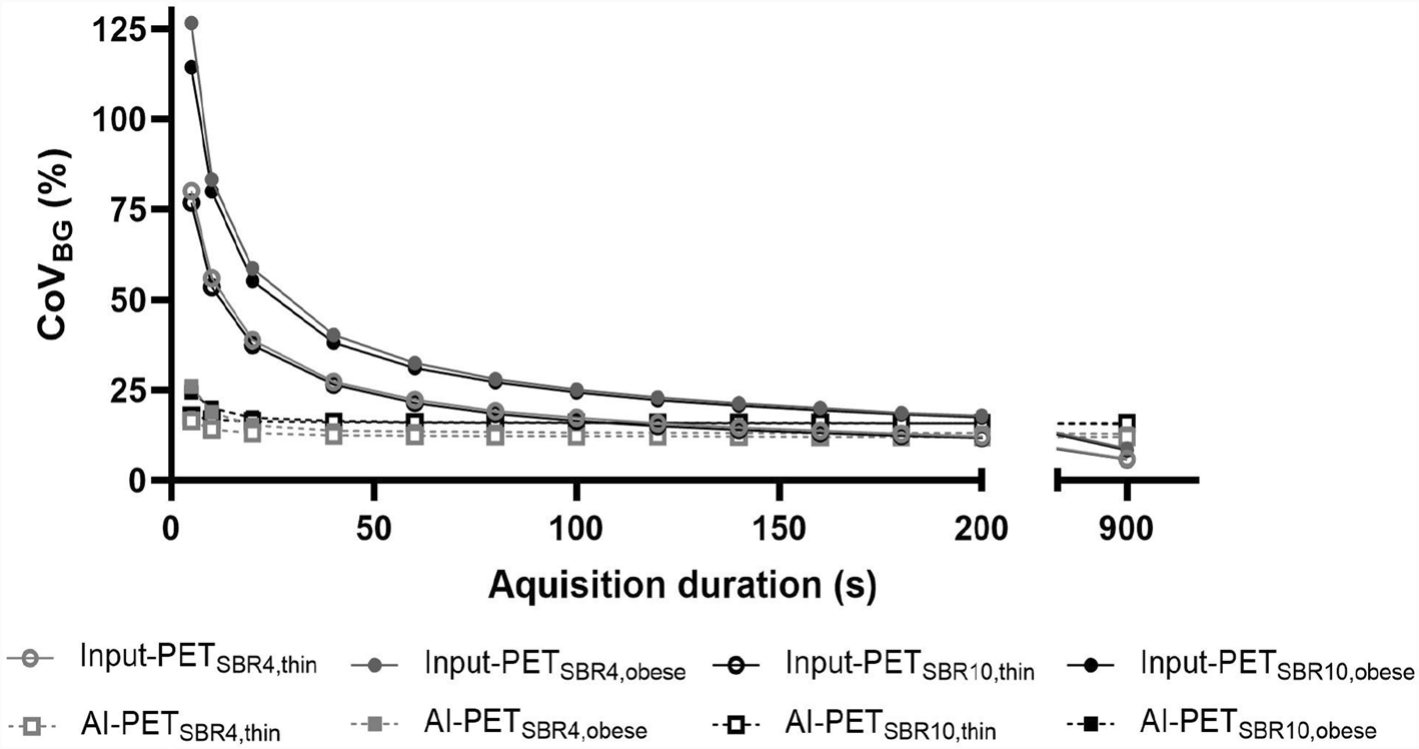
Image noise (CoV_BG_) of Input-PET and AI-PET scans as function of acquisition duration. Image noise (CoV_BG_) is shown as a function of acquisition duration (5 s, 10 s, 20 s to 200 s in steps of 20 s and 900 s) of Input-PET (solid line) and AI-PET scans (dashed line) for each of the four phantom setups (SBR4: gray circle; SBR10: black square; thin: open circle/square; obese: closed circle/square). The smaller image in the top right-hand corner shows the CoV_BG_ for a smaller range of 0 ≤ CoV_BG_ ≤ 27

**Fig. 4:**
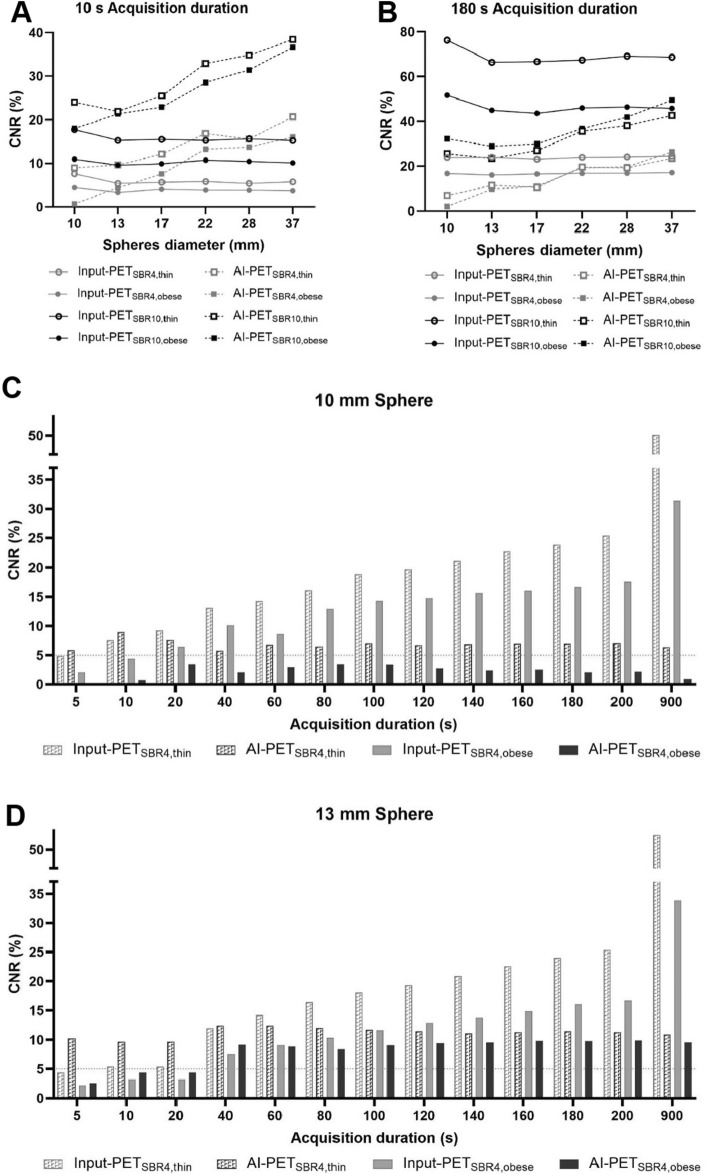
Contrast Noise Ratio (CNR) of Input-PET and AI-PET scans for each of the four phantom setups. Contrast Noise Ratio (CNR) of Input-PET (solid lines, circles) and corresponding AI-PET scans (dashed lines, squares) is shown for each of the four phantom setups (SBR4: gray; SBR10: black; thin: open circles/squares; obese: closed circles/squares). CNR values of the 10 s acquisition duration scans (**A**) and 180 s acquisition duration scans (**B**) are shown as a function of the inner sphere diameter ranging from 10 to 37 mm. CNR values of the sphere of the NEMA phantom with 10 mm (**C**), 13 mm (**D**) and 17 mm (**E**) inner diameter are shown as a function of acquisition duration (5 s, 10 s, 20 s to 200 s in steps of 20 s and 900 s)

Compared to the corresponding ground truth scan, image quality of the low-count Input-PET improved with increasing acquisition duration and became more and more similar to the ground truth scan: noise in the background compartment decreased and (especially small) lesions were better visible (Fig. 2). Consistently, CoV_BG_ decreased in Input-PET scans with increasing acquisition duration/image statistics and became more and more similar to the CoV_BG_ in the ground truth scan (Fig. 3 and supplementary Table S.4), the relationship follows a power law (r^2^ = 1 for all 4 setups). In addition, CNR increased with increasing acquisition duration in Input-PET, which indicates improving visibility of the spheres in scans featuring higher image statistics (Fig. 4).

Visual evaluation of Fig. 2 revealed that image quality of AI-PET scans was much better than that of the low-count Input-PET scans for very short acquisition durations ≤ 20 s: image noise was lower and the visibility of lesions was better, especially for small lesions. Consistenty, semi-quantitative evaluation of image quality revealed that the deep-learning neural network was very well able to reduce CoV_BG_ when applied to ultra-short Input-PET scans ≤ 60 s featuring very high noise levels (Fig. 3 and supplementary Table S.4). Even when Input-PET scans featured a CoV_BG_ of up to 126.63%, as for the SBR4, obese setup and 5 s scan time, the AI algorithm was able to strongly reduce the noise level to 26.0% (supplementary Table S.4).

Both in Input-PET_SBR4, thin_ and Input-PET_SBR4, obese_ scans, the two smallest spheres of the phantom featuring an inner diameter of 10 mm and 13 mm, respectively, were no longer detectable in ultra-short scans of 5 s acquisition duration (Fig. 2). Consistently, CNR of those two spheres was ≤ 5 in Input-PET_SBR4, thin_ with 5 s acquisition duration and in Input-PET_SBR4, obese_ scans with 5 s and 10 s acquisition duration, respectively (Fig. 4), indicating that lesions were no longer visible as of the Rose criterion [10]. In comparison, in AI-PET_SBR4, thin_ scans, even in ultra-short recordings as short as 5 s, all spheres were visible (Fig. 2) and CNR values were > 5 for all spheres (Fig. 4).

Although image quality of low-count Input-PET scans improved both visually and semi-quantitively with increasing acquisition duration, visual image quality of *AI-enhanced* AI-PET scans was similar in all AI-PET scans ≥ 40 s acquisition duration and therefore almost independent of the quality of the corresponding Input-PET scans (Fig. 2). Consistent with visual evaluation, CoV_BG_ was in a similar range in all AI-PET scans ≥ 20 s acquisition duration and similar to that of the corresponding ground truth Input-PET scan (Fig. 3 and supplementary Table S.4) Similarly, for each phantom setup, semi-quantitative evaluation of CNR revealed similar values in all AI-PET scans with ≥ 60 s acquisition duration of the corresponding Input-PET scans and much lower CNR values compared to the corresponding ground truth Input-PET scan (Fig. 4).

However, when acquisition duration of the Input-PET scans exceeded that of the corresponding ground truth scan, visual image quality of Input-PET was better than that of the corresponding AI-PET (Fig. 2). Consistently, in cases where Input-PET data were of very high statistics and subsequently of low CoV_BG_, the AI-algorithm generated AI-PET data of paradoxically increased CoV_BG,_ as for example for the SBR4, thin phantom setup and 900 s acquisition duration (supplementary Table S.4). In addition, in all Input-PET scans with scan durations ≥ 60 s, CNR values of all spheres were higher than in the corresponding AI-PET images (Fig. 4), indicating better visibility of lesions in Input-PET compared to corresponding AI-PET scans (Fig. 4). When analyzing each individual Input-PET dataset, CNR values were comparable between spheres of different sphere diameters. In comparison, CNR values decreased with decreasing sphere diameter in each AI-PET scan, indicating a deterioration of the visibility of lesions with decreasing lesion size (Fig. 4).

While in Input-PET_SBR4, thin_ and Input-PET_SBR4, obese_ scans with acquisition duration ≥ 20 s all spheres were visually detectable, the smallest sphere of 10 mm diameter was difficult or impossible to visually detect in all AI-PET_SBR4, obese_ and AI-PET_SBR4, thin_ scans, respectively, even for very long acquisition durations of 900 s (Fig. 2). Consistently, CNR of the 10 mm diameter sphere were far below 5 in all AI-PET_SBR4, obese_ scans (maximum: 3.45 in AI-PET_SBR4, obese_ of 20 s duration) and much lower in AI-PET_SBR4, thin_ compared to corresponding Input-PET_SBR4, thin_ scans for acquisition durations ≥ 20 s (Fig. 4).

AI-PET_SBR4, obese_ scans were conspicuous since CNR values of the two smallest spheres were not only < 5 for ≤ 20 s acquisition duration scans (as for the other three phantom setups), but CNR values of the two smallest spheres were < 5 in all scans of this scenario and therefore independent of the count statistics of the Input-PET (Fig. 4).

### Image-to-image comparison using image quality measurement metrics (SSIM, PSNR, MAE)

In accordance with the original paper [5] and as in other studies on the use of AI for image enhancement [3], also in the phantom validation performed here the application the AI Algorithm to Input-PET data with short acquisition duration well restored structural information and enhanced image quality in AI-PET images compared to low count Input-PET data, as assessed semi-quantitively by an increase in SSIM and PSNR and a decrease in MAE (Table 1 and supplementary Tables S.7 and S.8). As summarized in Table 1, the phantom-based validation resulted in similar SSIM values as in the clinical validation in [5]. Other AI-based algorithms for image enhancement of low-dose whole-body PET images also revealed similar SSIM values when evaluated on clinical data [3].

**Table 1:** Image-to-image metric Structural Similarity Index Measure (SSIM) comparing Input-PET or AI-PET scans with the respective ground truth scan

Acqusition duration (s)	SBR4, thin		SBR4, obese		SBR10, thin		SBR10, obese	
	Input-PET	AI-PET	Input-PET	AI-PET	Input-PET	AI-PET	Input-PET	AI-PET
5	0.978	0.998	0.964	0.991	0.984	0.998	0.968	0.995
10	0.99	0.998	0.981	0.998	0.993	0.998	0.985	0.998
20	0.995	0.997	0.992	0.998	0.997	0.997	0.993	0.998
40	0.998	0.996	0.996	0.998	0.999	0.997	0.997	0.998
60	0.999	0.995	0.998	0.998	0.999	0.997	0.998	0.998
80	0.999	0.995	0.999	0.998	1	0.997	0.999	0.998
100	1	0.995	0.999	0.998	1	0.997	0.999	0.998
120	1	0.995	0.999	0.998	1	0.997	1	0.998
140	1	0.995	1	0.998	**1**	0.997	1	0.998
160	1	0.995	1	0.998	1	0.997	1	0.998
180	**1**	0.995	1	0.998	1	0.997	1	0.998
200	1	0.995	**1**	0.998	1	0.997	**1**	0.998
900	1	0.995	0.999	0.997	1	0.997	0.999	0.998

Direct image-to-image comparison of each Input-PET and AI-PET scan, respectively, with the respective ground truth scan as of the Structural Similarity Index Measure (SSIM) for the different acquisition durations and for each of the four phantom setups (SBR4, thin: first column; SBR4, obese: second column; SBR10, thin: third column; SBR10, obese: fourth column). For each phantom setup, the Input-PET scan, which was considered as reference ground truth scan, is highlighted in bold. SSIM was calculated using a mask for the entire phantom.

The validation using phantom data presented here yielded comparable values for the validation of the AI algorithm using clinical data in [5], where median SSIM was ~ 0.982 in the Extreme-Input-PET and ~ 0.993 in AI-PET data. SSIM of all AI-PET data was very similar and ranged between 0.991 and 0.998.

**Fig. 5:**
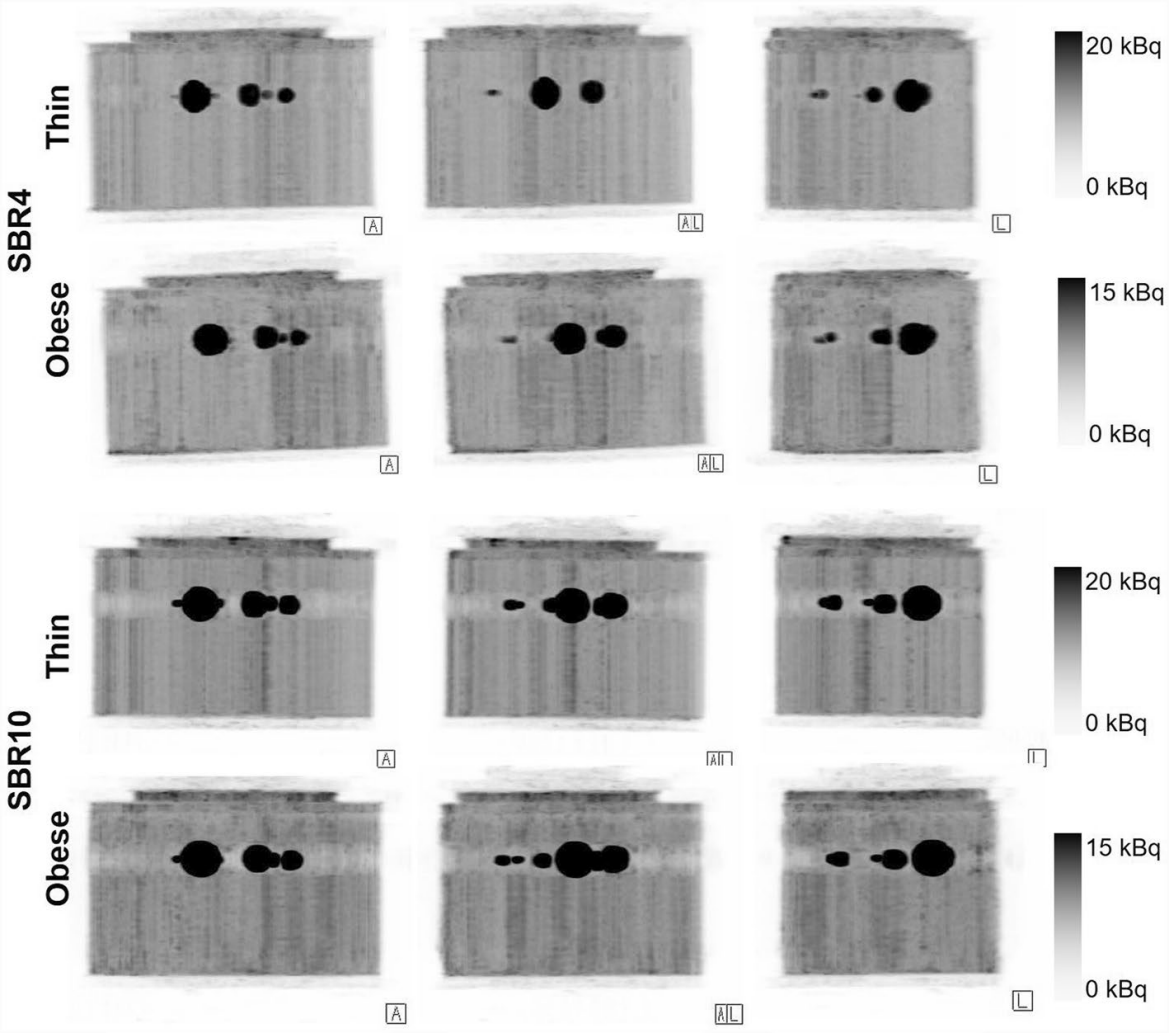
Maximum intensity projection of AI-PET scans. Maximum intensity projection of the AI-PET scans of the four phantom setups and at 180 s acquisition duration in axial (colum 1), axial/lateral (colum 2), and lateral view (colum 3)

All AI-generated image data featured strip-shaped artifacts, as illustrated in Fig. 5 representatively for the 180 s acquisition durations. This is probably the main reason why all AI-PET scans showed an increased MAE, even when PET scans of high image quality served as AI-input like the ground truth data or Input-PET scans with even longer acquisition durations.

Compared to the clinical validation presented in [5], the phantom validations provide further information: For Input-PET data, SSIM and PSNR decreases and MAE increase with decreasing acquisition duration, which means a change of structural information, deterioration in image quality and noise increase as compared to the ground truth data (Table 1 and supplementary Tables S.7 and S.8). Such an image deterioration is to be expected by the decreasing image statistics. However, when acquisition duration was sufficiently long (≥ 140 s) or even longer than the ground truth data, structural information remained uniform and unchanged in Input-PET data (which is reflected in a SSIM of 1 in those Input-PET data, Table 1). However, an application of the AI algorithm to Input-PET data with higher acquisition duration than the ground truth data (resulting in no change of structural information and an unchanged SSIM of 1 in the Input-PET data) resulted in a slight decrease of SSIM, strong decrease in PSNR and increase in MAE in AI-PET compared to ground truth scans, meaning a deterioration of image quality and slight modification of structural information compared to the ground truth data.

### Quantitative accuracy: recovery coefficients and SUV

**Fig. 6:**
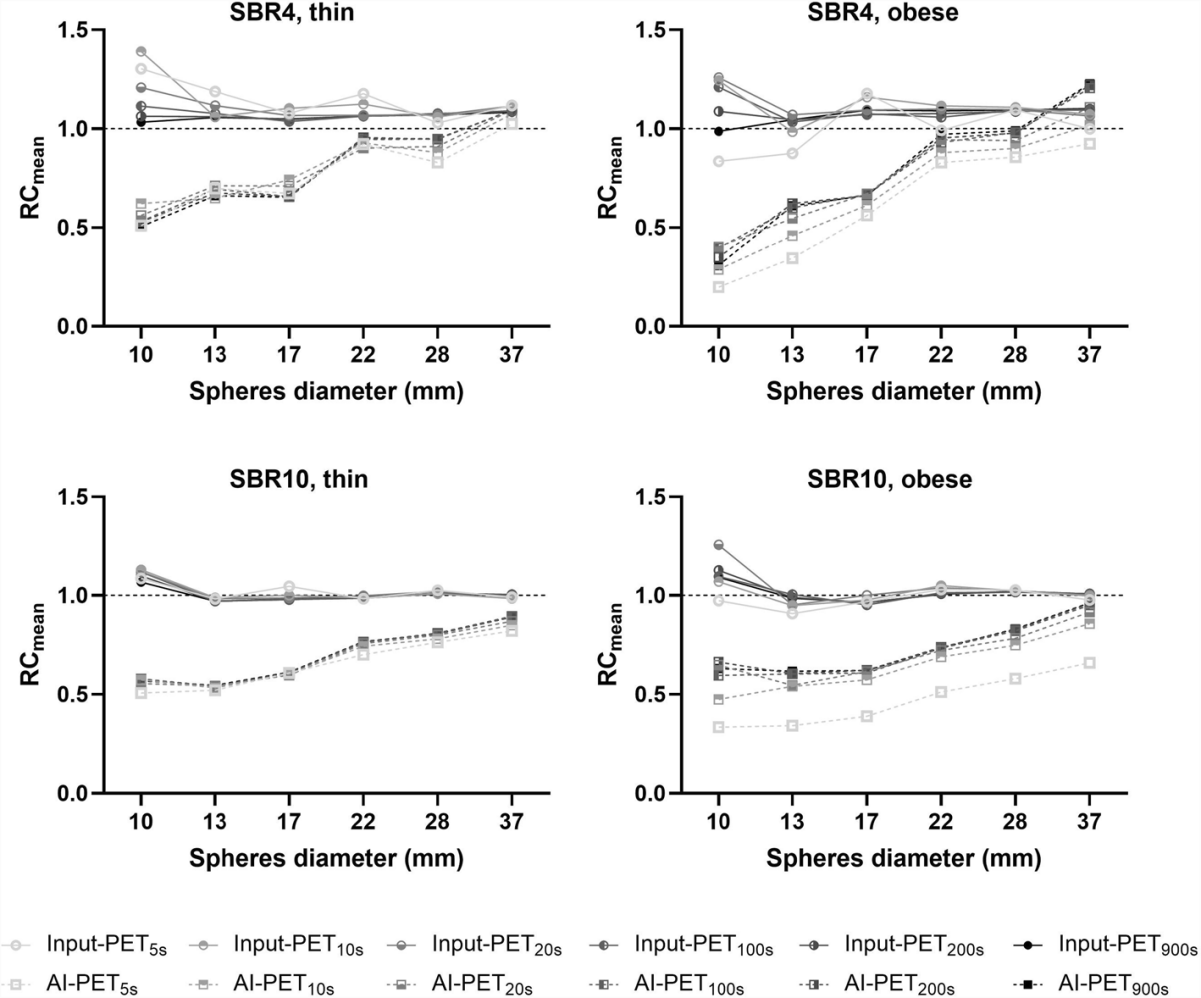
Mean Recovery Coefficient (RC_mean_) of Input-PET and AI-PET scans for each of the four phantom setups. The Mean Recovery Coefficient (RC_mean_, determined as the measured SUV_mean_ relative to the actual activity concentration in the phantom sphere) is shown as a function of the inner sphere diameter ranging from 10 to 37 mm of Input-PET (solid lines, circles) and AI-PET scans (dashed lines, squares) for different acquisition durations (5 s to 900 s: light gray to black) for each of the four phantom setups (SBR4: upper row; SBR10: lower row; thin: left column; obese: right column)

For spheres with inner diameter > 10 mm and acquisition durations > 10 s, RC_mean_ values were similar in all Input-PET scans and ranged between 0.95 and 1.12 (Fig. 6). In Input-PET, RC_mean_ of the smallest sphere of 10 mm diameter fluctuated a bit more and reached values between 0.84 (Input-PET_5s, SBR4, obese_) and 1.39 (Input-PET_10s, SBR4, thin_). Comparing AI-PET with ground truth or with all other Input-PET scans for corresponding sphere diameter and the four phantom setups, RC_mean_ values were much lower in all AI-PET datasets except for the largest sphere of 37 mm diameter. While RC_mean_ values were independent of sphere diameter in the respective ground truth scans, RC_mean_ values strongly decreased with decreasing sphere diameter in all AI-PET scans. The maximum underestimation of SUV_mean_ occurred in AI-PET_5s, SBR4, obese_: RC_mean_ was as low as 0.2 for the 10 mm inner diameter sphere.

**Fig. 7:**
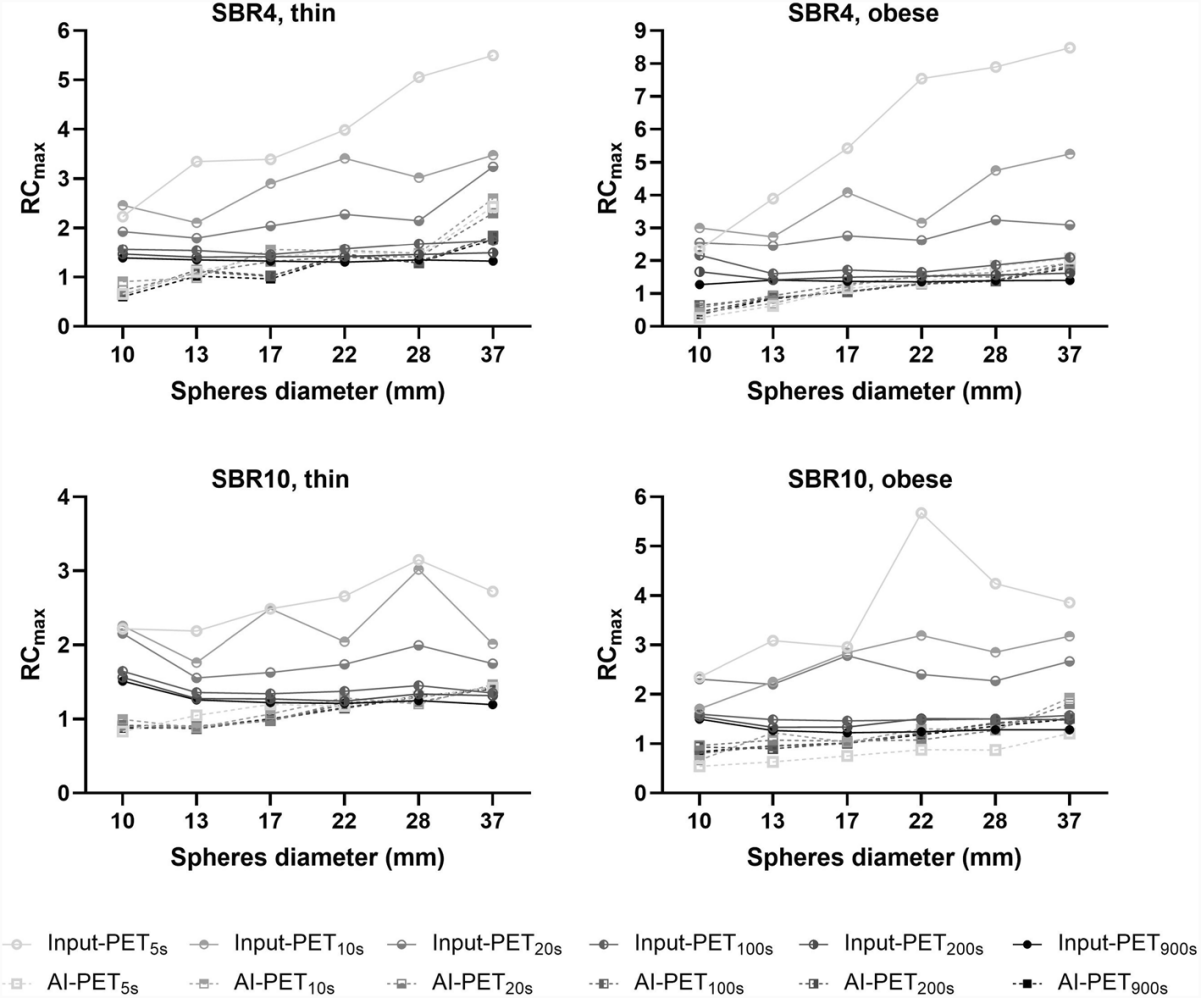
Maximum Recovery Coefficient (RC_max_) of Input-PET and AI-PET scans for each of the four phantom setups. Maximum Recovery Coefficient (RC_max_, determined as the measured SUV_max_ relative to the actual activity concentration in the phantom sphere) as function of the inner sphere diameter ranging from 10 to 37 mm for Input-PET (solid lines, circles) and AI-PET scans (dashed lines, squares) for different acquisition durations (5 s to 900 s: light gray to black) for each of the four phantom setups (SBR4: upper row; SBR10: lower row; thin: left column; obese: right column)

As of Fig. 7, RC_max_ values were much lower in AI-PET compared to the respective ground truth or all other Input-PET datasets for all phantom setups and for all spheres featuring an inner diameter ≤ 22 mm. While RC_max_ values increased with decreasing acquisition time/statistics in Input-PET scans, RC_max_ values were similar in all AI-PET scans of differing acquisition durations. RC_max_ values strongly decreased with decreasing sphere diameter in each of the AI-PET scans, while no such dependence of RC_max_ on sphere diameter occurred in ground truth scans (but in Input-PET data of low statistics). Absolut differences in SUV_max_ between Input-PET and corresponding AI-PET were comparable for differing acquisition durations but increased with decreasing sphere diameter and ranged between mean ± SD of 1.55 ± 0.42 for smallest spheres of the SBR4 and obese phantom scans and 0.02 ± 1.36 for spheres of 37 mm diameter in the SBR4 and thin phantom scan.

As shown in the supplementary Tables S.5 and S.6, the validation of the semi-quantitative accuracy of the AI algorithm using phantom data (at SBR4 and thin patient setup) yielded similar values as using clinical data in [5] for the absolute difference of SUV_mean_ and SUV_max_ values between AI-PET and the respective ground truth scan, respectively, when analyzing all lesions or more specifically only lesions in lymph nodes. A higher sphere-to-background contrast ratio of 10 in the phantom validation resulted in higher absolute SUV_mean_ and SUV_max_ differences, respectively, than the validation using clinical data presented in [5]. Except for ultrashort scans with acquisition durations ≤ 20 s (which corresponds to a ≤ 10-times reduced acquisition duration), the SUV differences in all AI-PET scans of different acquisition durations were of a similar order of magnitude. In contrast, the acquisition duration of the ground truth Input-PET scans can be shortened to an acquisition duration of about half of that of the ground truth Input-PET scans without introducing any SUV_mean_ and strong SUV_max_ differences, respectively. Even for Input PET scans with acquisition durations of about 1/3 of that of the ground truth scans and longer, the SUV differences compared to ground truth scans were smaller than those of AI-generated AI-PET scans.

### AI versus Gaussian filter

**Fig. 8:**
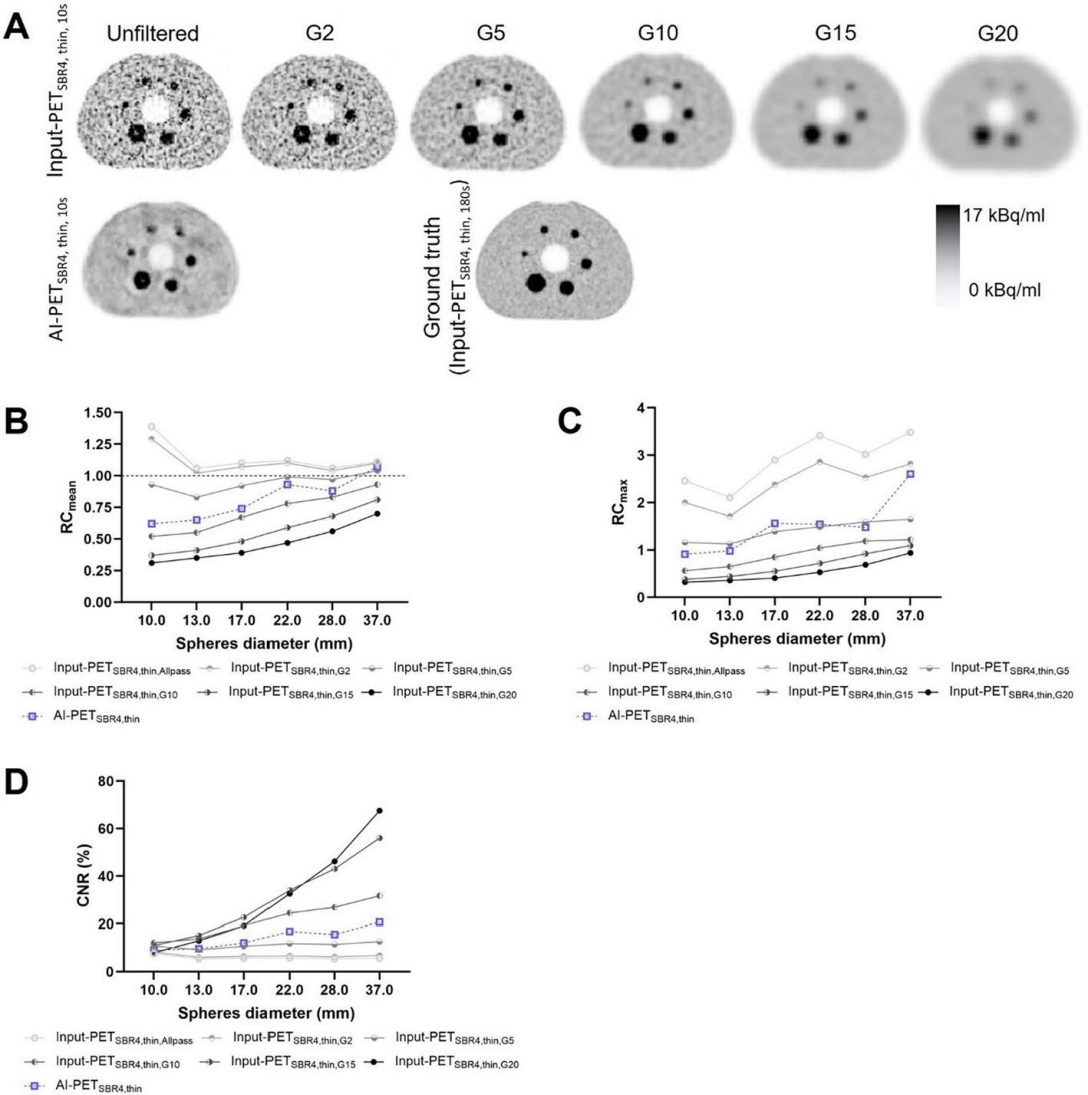
Qualitative and semi-quantitative comparison of unfiltered and post-filtering Input-PET scans and the corresponding AI-PET. Qualitative (**A**) and semi-quantitative (**B**–**D**) comparison of the impact of post-filtering of the Input-PET_SBR4, thin_ scans with 10 s acquisition duration as well as the AI-PET_SBR4, thin_ scan corresponding to the unfiltered Input-PET scan. Post-filtering of the Input-PET_SBR4, thin_ scan was applied using a Gaussian filter with 2 mm FWHM (G2), 5 mm FWHM (G5), 10 mm FWHM (G10), 15 mm FWHM (G15), and 20 mm FWHM (G20, A). For qualitative comparison in A, all scans are shown at the same axial position and using the same color scale. For semi-quantitative comparison, Mean and Maximal Recovery Coefficient (RC_mean_/RC_max_, determined as the measured SUV_mean_/SUV_max_ relative to the actual activity concentration in the phantom sphere) are shown in B/C as a function of the inner sphere diameter ranging from 10 to 37 mm for Input-PET without and with G2 to G20 filtering (solid lines, circles) and AI-PET_SBR4, thin_ scans (dashed line, blue). Contrast Noise Ratio (CNR) is shown in D as a function of the inner sphere diameter ranging from 10 to 37 mm for Input-PET without and with G2 to G20 filtering (solid lines, circles) and AI-PET_SBR4, thin_ scans (dashed line, blue)

Figure 8 and Table 2 compare unfiltered and post-filtered Input-PET_SBR4, thin_ scans with 10 s acquisition duration with the AI-PET_SBR4, thin, 10 s_ scan and the ground truth scan, respectively.

**Table 2:** Semi-quantitative comparison of unfiltered and post-filtering SBR4,thin,10 s-Input-PET scans with respective ground truth scan

		CoV_BG_	SSIM	PSNR	MAE	∆SUV_mean_	∆SUV_max_
Input-PET_SBR4, thin,10 s_	Unfiltered	79.4	0.990	8.11	0.534	0.33 ± 0.48	5.69 ± 2.05
	G2:	46.8	0.993	9.64	0.451	0.26 ± 0.32	3.64 ± 1.75
	G5:	23.1	0.998	20.29	0.247	0.49 ± 0.27	0.61 ± 0.53
	G10:	7.9	0.999	51.48	0.169	0.71 ± 0.58	1.2 ± 1.16
	G15:	3.7	0.999	96.11	0.173	2.03 ± 0.63	3.13 ± 0.99
	G20:	2.4	0.998	126.52	0.186	2.41 ± 0.54	3.71 ± 0.79
	AI-PET	13.99	0.998	35.40	0.235	1.01 ± 0.67	1.54 ± 1.53
Ground truth (Input-PET_SBR4, thin, 180 s_)		12.89	1	33.53	0	0 ± 0	0 ± 0

Semi-quantitative comparison of the impact of post-filtering of the Input-PET_SBR4, thin_ scans with 10 s acquisition duration. Post-filtering of the Input-PET_SBR4, thin, 10 s_ scan was applied using a Gaussian filter with 2 mm FWHM (G2), 5 mm FWHM (G5), 10 mm FWHM (G10), 15 mm FWHM (G15), and 20 mm FWHM (G20). For semi-quantitative comparison, Image noise (CoV_BG_) of each scan was determined. Direct image-to-image comparison between the unfiltered and each post-filtered scan, respectively, and the ground truth scan was done as of the Structural Similarity Index Measure (SSIM), Peak Signal to Noise Ratio (PSNR), and Mean Absolute Error (MAE). Mean and standard deviation of the sphere-based difference in SUV_mean_ (∆SUV_mean_) and SUV_max_ (∆SUV_max_), respectively, were calculated comparing each scan with the ground truth scan.

In summary, the AI algorithm improved image quality better than when a Gaussian filter was applied to the data, with the FWHM of 5 mm to 10 mm filter performing best when considering the different Gaussian filter sizes. In detail, a visual analysis revealed an almost as good image quality (low background noise and good recognizability of the spherical structures) in ground truth and AI-PET_10s scans_ and only little deterioration in the 5 mm or 10 mm FWHM filtered scans, while it was worse in 2 mm, 15 mm and 20 mm FWHM post-filtered scans.

Semi-quantitative analyses confirmed that CoV_BG_ was in the same order of magnitude in AI-PET_10s_ and ground truth scans and only slightly lower in the Input-PET_10s, G10_ scan, but higher in the Gaussian-filtered images when using a lower filter size than 10 mm (Table 2). The image quality measurement metrics analyses confirmed a very high structural similarity (SSIM ≥ 0.998) and the smallest differences in PSNR and MAE when comparing the ground truth scan with the AI-PET, 5 mm, or 10 mm Gaussian filtered images, while it was worse in the Gaussian filtered images using a FWHM of 2 mm, 15 mm or 20 mm (Table 2). Due to the strong reduction of image noise by post-filtering, CNR decreased significantly for the smallest spheres as the filter size increased (Fig. 8). As CNR is a measure of lesion visibility, this confirmed the visual impression that the small lesions became blurred with increasing filter size and were increasingly difficult to distinguish from the ground.

While compared to the ground truth data the image quality was better in the AI-PET scan than in the post-filtered data, qualitative accuracy was worse. Underestimation of SUV_mean_ increased with increasing Gaussian filter size (increase in SUV_mean_ difference and decrease in RC_mean_ with values lower 1, Fig. 8 and Table 2) and SUV inaccuracy was higher in AI-PET compared to post-filtered scans when a FWHM of 2 mm to 10 mm FWHM filter size was used. SUV values were only consistent between spheres of different sizes in Input-PET_10s, G2_ and Input-PET_10s, G5_ scans, but not in the AI-PET scan or stronger filtered images.

## Discussion

In this study, a phantom-based validation of an deep-learning based denoising algorithm presented in [5] was performed. This study revealed that the presented AI algorithm is well suited to improve image quality in ultra-low count PET scans with acquisition durations ≤ 20 s by reducing image noise, increasing CNR as a measure of the visibility of lesions and improving structural characteristics of lesions. Therefore, the presented algorithm fulfills its task very well for the scenario of ultra-short PET scans, for which it was trained for. However, the AI-generated PET scans are not suitable for semi-quantitative SUV analysis of smaller lesions with a diameter ≤ 17 mm since SUV was strongly underestimated for all tested scenarios in small lesions. In addition, the extent of SUV underestimation depended on lesion size, the proportion of attenuation (thin vs. obese patients) and the lesion-to-background contrast ratio, which further limits the applicability of AI-generated PET scans for SUV quantification.A comparison of the performance of the neural network with post-processed PET images using a Gaussian filter as frequently used denoising method in clinical routine revealed that the neural network is a better choice in order to improve visual image quality, but at the same time degrades SUV accuracy to a greater extent than post-filtering.

A phantom-based validation of an AI-based algorithm enables a comprehensive assessment of the performance, limitations, and generalizability of such an algorithm for PET image enhancement. It allows for controlled testing of the performance of the algorithm under different and reproducible conditions with well-known setup and measurement conditions, such as lesion size or activity concentrations in the phantoms. In this study, the performance of an AI-based denoising algorithm was examined using Input-PET data featuring a broad spectrum of image statistics ranging from ultra-low to ultra-high count PET data as acquisition duration and thus counting statistics is directly related to image quality, CNR and detectability of lesions [1, 8, 12]. In addition, the performance of the presented deep-learning neural network in terms of image quality enhancement was tested for different sphere-background contrast ratios and for different patient weight setups of the NEMA PET body phantom, which have both also been shown to affect CoV_BG_ and image quality [1] and can affect the visibility of small lesions. Wrapping the phantom with cooling packs increased the proportion of attenuation and scatter and resulted in a 45% to 48% reduction of the detected number of true events, which is comparable to a ~ 50% reduction of the acquisition time. Therefore, the obese test scenario allowed a performance analysis under even more extreme conditions as a further reduction of the acquisition duration lower than 5 s is not possible when using a Biograph Vision 600 PET/CT scanner. The known activity concentration and the presence of spheres of different sizes in the phantom also allowed a detailed analysis of the quantitative accuracy of the AI algorithm.

For a comparison of the AI-generated image data with ground truth data reflecting the quality of PET data on which the algorithm was trained on and against which the AI-generated PET data were already clinically validation in [5], ground truth Input-PET data were determined for each phantom setup. Comparisons of the AI-generated PET images with the corresponding ground truth data revealed that the AI algorithm was very well able to improve image quality of very short and subsequently low-count Input-PET data. Noise levels in almost all AI-PET scans were in the range of the corresponding ground truth scans and almost independent of the CoV_BG_ level of the statistics of the input data. Only in the most extreme test scenarios of ultra-short (< 20 s) PET scans of the obese phantom setups and SBR of 4, where true count rates were as low as 2.5% (5 s) compared to that of the corresponding ground truth dataset(supplementary Table S.3), the deep-learning algorithm was slowly reaching its limits and noise levels were about double as high as in less extreme test conditions. However, compared to noise levels in Input-PET, the algorithms still achieved a very good result in terms of noise reduction. However, under such extreme conditions, the algorithm was no longer able to distinguish the two smallest spheres from noise, such that only spheres with a diameter ≥ 17 mm (which corresponds to a spherical lesion volume of ≥ 20.6 ml) were visible in the AI-PET data.

However, when the deep-learning algorithm was applied to Input-PET data of acquisition durations longer than the ground truth data featuring accordingly higher image statistics (Fig. 1 supplementary Table S.3), better image quality and very low CoV_BG_ levels of 6% to 9% (Fig. 3), the deep-learning algorithm degraded image quality and generated AI-PET scans featuring higher noise levels compared to higher-count Input-data. The algorithm seems not to recognize when the noise level in the input data is less than its learned optimum, which could indicate a variant of the Clever Hans effect and highlights the need to rigorously test the boundary condition in which AI is properly functioning [13, 14].

In addition to incorporating an analysis of the input–output noise level, the performance of the deep-learning neural network might also be improved by training it on data with higher statistics and subsequent lower CoV_BG_, like PET scans of longer acquisition duration, higher injected dose values as reported to be standard in the USA and Canada (6 MBq/kg: [1]) or data acquired with standard acquisition time and dose in Europe but using an ultra-sensitive PET scanner, such as long axial field of view PET scanner. This would allow all departments that only have an older generation PET scanner available to optimize image quality, as it would be achievable with newer generation scanner, as already shown for deep-learning-based algorithms that upscales PET scans from short- to long-axial field of view scanner quality [15]. It would be very interesting to validate in future studies whether training this and similarly structured AI algorithms on ground truth data with better image quality improves performance.

The validation also showed that the algorithm might only be used after precise knowledge of its limitations and the dependency of the accuracy of SUV quantification on lesion size, the proportion of attenuation (thin vs. obese patients) and the lesion-to-background contrast ratio. For very large lesions with a diameter of about 37 mm (which corresponds to a volume of 26.5 cm^3^), the SUV-values were accurately recovered in AI-PET scans even though the true count level of the Input-data was reduced to 1/35 and thus 1 million true counts. This suggests that the AI-generated data are suitable for a quantitative analysis of a homogeneous tracer accumulation in larger organs or lesions. However, SUV values of smaller spheres with diameters ≤ 17 mm (≤ 2.57 cm^3^ in spherical volume) were strongly underestimated, and underestimation increased with decreasing lesion size. This is a severe limitation in respect the applicability of the algorithm in clinical routine since the SUV value for lesions of typical size is then underestimated. Lesion sizes up to a lowest / mean size of 0.2 cm^3^ / 4.6 cm^3^ have been described as typical for patients with lung cancer [12], which corresponds to lesions with a diameter of 7.26 mm / 20.634 mm assuming a spherical shape. This is the range were the algorithm might strongly underestimate SUV values.

The quantitative inaccuracy and SUV underestimations, especially for smaller spheres, seemed not to be a result of ultra-low count input-data as assumed in [5], but also occurred to a comparable extent when the deep-learning algorithm was applied to PET data of shorter and longer acquisition durations than the respective ground truth datasets, respectively.

This contradicts the statement in [5] that the SUV differences are most likely due to the very short acquisition durations. Rather, the results presented here indicate a systematic error of the deep-learning algorithm and a structural problem of the applied GAN. The fact that, compared to the ground truth scan, SUV differences were small for short (but not ultrashort) Input-PET scans, and lower than for AI-generated AI-PET scans, suggests that for absolute SUV quantification, Input-PET scans with poor (but not extremely low) image statistics are more suitable than AI-generated AI-PET scans. These findings illustrate once again that a systematic analysis of an AI algorithm is important and that phantom-based validations are very well suited for this purpose, as they allow the performance of the AI algorithm to be analyzed on the basis of different acquisition conditions, such as acquisition duration or degree of tracer uptake in lesions (which corresponds to SBR in phantoms).

If a quantitative assessment is planned, the SubtlePET software seems to be a better choice for image optimization of PET scans featuring 33% or 50% reduced count statistics than standard dose and scan duration PET scans acquired compared to the denoising deep-learning algorithm validated here. A small underestimation of SUV_mean_ and SUV_max_ in the range of 5% was reported for the SubtlePET software as well, but to a much smaller extent than for the validated denoising algorithm in this study and despite similar CoV_GB_ values in the SubtlePET-generated PET scans [1]. However, the denoising deep-learning algorithm validated here was not developed for PET scans featuring 33% to 50% reduced count statistics, but for PET scans with significantly lower counts. Nevertheless, an integration of optimization steps as implemented in SubtlePET [1] may help to correctly recover SUV values while maintaining the excellent very good noise reduction properties to a level comparable to full-time and -dose PET images.

In addition to changes of the AI architecture, the quantitative accuracy of the AI algorithm in respect to a better prediction of the actual activity concentration might be improved by training it on unfiltered PET data instead of using a 4 mm Gaussian filter as in [5]), as SUV have shown to decrease with increasing size of the Gaussian post-filter especially for smaller spheres (Fig. 8 and [16, 17]). However, the underestimation of the SUV values in AI-PET scans cannot be explained solely by training the algorithm on post-processed data using a Gaussian filtered but seems to be a systematic error. Although the extent of SUV underestimation increases with increasing filter size and Gaussian filtering causes the SUV recovery to depend on the sphere size, an exclusive filtering of the input PET data with Gauss 5 mm resulted in a better recovery of the SUV_mean_ values. Nevertheless, the CoV_BG_ in the AI-PET data was lower than in 2 mm and 5 mm Gaussian postfiltered scans, such that the denoising algorithm is better suited for noise reduction than pure filtering of the data with Gauss ≤ 5 mm. And even with post-processing with larger Gaussian filters, the algorithm achieved a better compromise between noise reduction while at the same time minimizing SUV underestimation. Therefore, the AI algorithm evaluated here performed better than Gaussian filtering and should therefore be preferred to pure (Gaussian) filtering if noise suppression alone is to be achieved. Consistently, also other studies have shown that the inclusion of AI-based denoising approaches achieves better results than the pure conventional Gaussian post-filtering method [18–20], as the images not only have less noise, but also retain all detailed features. Nevertheless, Gaussian post-filtering is still considered the clinical standard and is very frequently used in clinical routine.

The strip-shaped artifacts visible in the AI-generated image data as well as the very poor visibility of the two smallest sphere in the AI-PET_SBR4, obese_ datasets are most likely due to the 2.5D and slice-wise approach of the deep-learning neural network and the batch size of 4 slices. Especially when the contrast is low, the contrast may not be sufficient for the deep-learning algorithm to recognize small lesions as such and perceives it as noise. This is a strong limitation of the algorithm, as small lesions with a size of about 10 mm may be hidden in AI-PET scans. Likewise, nodal lymphoma lesions have been described to typically feature sizes of ≥ 10 mm [21]. A 3D approach for denoising of low count PET data might therefore be a better choice, as presented to be suitable for improvement of image quality, reduction of image noise and quantitative while maintaining quantitative accuracy for lesions larger 1 cm^3^ in volume for PET scans of lung cancer patients [12]. Future studies are desirable to validate the performance improvement of this and similar 2.5D algorithms by modifying to a fully 3D framework, especially with respect to a mitigation of the strip-like artifacts and an improvement of detectability of small lesions.

In addition, a modification of the GAN-based deep learning method and application of other approaches, such as Convolutional Neural Networks, Vision-Transformer or diffusion-based architectures, which have also shown potential for enhancing PET image quality [22–24], could improve the performance of the AI algorithms. A comparative evaluation of these techniques would be beneficial for future research to expand the scope of AI in PET imaging.

A further improvement of the deep-learning neural network could be achieved by integrating deep convolutional networks during the reconstruction process itself and taking PET sinograms as input [25] or by training the network on multi-center datasets instead of training exclusively on datasets generated in a single center using the same device [26].

Despite the shortcomings of the tested AI-based denoising approach, the denoising algorithm could be very helpful in terms of qualitative image enhancement of noisy data, where absolute quantification and the detection of very small lesions is of less relevance compared to dose or acquisition time reduction. Such applications would be screening [27], pediatric examinations [4], dynamic imaging and the reduction of motion artefacts, or gated PET scanning [22].

## Limitations

Images of phantoms cannot depict the reality as in the patient. For example, phantoms feature a background compartment with a homogeneous activity concentration, such that image noise is solely a measure of image quality. In comparison, activity concentrations in human reference organs such as the liver can be physiologically heterogeneous. Since the deep-learning neural network was trained on more heterogeneous patient data, it speaks for the quality of the algorithm that it achieves good results even when applied to clinically more unusual phantom data. Another limitation of this study is that no physician reading of the AI-generated results was carried out, as such an assessment had already been carried out for clinical data in [5]. Third, the smallest sphere of the NEMA PET body phantom featured an inner diameter or volume of 10 mm or 0.52 ml. An analysis of the detectability and quantitative accuracy of smaller lesions could therefore not be performed with this phantom.

## Conclusions

In this study, the performance and potential limitations of a deep-learning neural network trained to denoise ultra-short PET images was evaluated using phantom scans. The systematic evaluation of the performance of the deep-learning algorithm under defined conditions revealed that the deep-learning network performs very well in improving image quality and educting image noise in ultra-low count PET scans. However, in respect to SUV accuracy, the AI algorithm must be used with good knowledge of its performance and limitations. SUV values were strongly underestimated for small lesions with a spherical volume ≤ 2.57 cm^3^, while the quantitative accuracy was accurate for large lesions ≥ 37 mm in diameter. In addition, likely due to the 2.5D approach of the algorithm, it might not recognize smaller lesions ≤ 13 mm in diameter (which corresponds to a sphere volume of 9.2 ml) as such but considers it as noise when applying to ultra-low count PET scans, like PET scans with an acquisition duration of ≤ 20 s, or when applying it to lower contrast images ≤ 4. In those cases, image quality of AI-PET scans might not be good enough for visual assessment of smaller lesions. In comparison to a standardized image post-processing using Gaussian post-filter approaches, the AI algorithm is better suited to improving image quality, but at the same time significantly reduces the quantitative accuracy of the images, especially of small lesions.

## Supplementary Information

Supplementary file 1.

## Data Availability

The datasets used and/or analyzed during the current study are available from the corresponding author on reasonable request.

## Abbreviations

AI: Artificial intelligence
PET: Positron emission tomography
CT: Computed tomography
AI-PET: AI-enhanced PET images
GAN: Generative adversarial network
Input-PET: Initial PET/CT scan of the NEMA PET body phantom
SBR: Sphere-to-background activity concentration ratio
FWHM: Full width at half maximum
VOI: Volume of interest
FoV: Field of view
CoV_BG_: Image noise
RC_mean_: Mean recovery coefficient
RC_max_: Maximum recovery coefficient
SUV_mean_: Mean standardized uptake value
SUV_max_: Maximum standardized uptake value
CNR: Contrast-to-noise-ratio
SSIM: Structural similarity index Metrics
PSNR: Peak signal-to-noise ratio
MAE: SUV-based mean absolute error
