# Supplementary material for: External phantom-based validation of a deep-learning network trained for upscaling of digital low count PET data
Source: EJNMMI Phys. 2025 Apr 16;12:38. doi: 10.1186/s40658-025-00745-4 (PMC12003253; doi:10.1186/s40658-025-00745-4)
Supplement: Supplementary file 1 [file 40658_2025_745_MOESM1_ESM.pdf]

Supplemental Material

to Manuscript with Title

External phantom-based validation of a deep-learning network trained for upscaling of digital low count PET data

Supplementary Table S.3: Image statistics of each Input-PET scan.

Image statistics of each Input-PET scan and phantom setup (SBR4, thin: first column; SBR4, obese: second column; SBR10, thin: third column; SBR10, obese: fourth column) specified as the detected number of true events per scan duration in Mio counts and are given for each acquisition duration ranging from 5 s to 900 s. For each phantom setup, the Input-PET scan, which was considered as reference ground truth scan, is highlighted in gray.

**Acqusition duration (s)**	**SBR4, thin**	**SBR4, obese**	**SBR10, thin**	**SBR10, obese**
**5**	1,00	0,54	1,25	0,65
**10**	2,00	1,09	2,50	1,31
**20**	4,00	2,18	5,00	2,61
**40**	8,00	4,35	9,99	5,22
**60**	11,99	6,52	14,97	7,83
**80**	15,98	8,68	19,94	10,43
**100**	19,95	10,84	24,90	13,02
**120**	23,91	12,99	29,84	15,62
**140**	27,87	15,15	34,77	18,20
**160**	31,82	17,30	39,70	20,78
**180**	35,76	19,44	44,61	23,36
**200**	39,69	21,58	49,51	25,93
**900**	172,33	93,78	215,00	112,69

Supplementary Table S.4: Image noise (CoV_{BG}) of Input-PET and AI-PET scans as function of acquisition duration.

Image noise (CoV_{BG}) in Input-PET and AI-PET scans is shown for the different acquisition durations and for each of the four phantom setups (SBR4, thin: first column; SBR4, obese: second column; SBR10, thin: third column; SBR10, obese: fourth column).

Acqusition duration (s)	SBR4, thin		SBR4, obese		SBR10, thin		SBR10, obese	
	Input-PET	AI-PET	Input-PET	AI-PET	Input-PET	AI-PET	Input-PET	AI-PET
5	80.10	16.46	126.63	26.00	76.83	17.77	114.37	24.28
10	55.92	13.99	83.30	18.78	53.46	16.75	80.05	20.00
20	38.84	13.09	58.61	15.24	37.44	16.32	55.22	17.23
40	27.29	12.40	40.23	13.65	26.44	16.13	38.04	16.41
60	22.26	12.27	32.50	13.45	21.40	15.98	30.99	16.09
80	19.09	12.22	28.04	13.35	18.38	15.89	27.11	16.03
100	17.27	12.18	25.06	13.21	16.43	15.90	24.36	15.95
120	15.73	12.14	22.96	13.16	14.96	15.86	22.19	15.83
140	14.55	12.08	21.25	13.11	13.91	15.85	20.62	15.81
160	13.67	11.99	19.93	13.09	13.03	15.84	19.33	15.79
180	12.89	11.95	18.57	13.08	12.33	15.81	18.30	15.77
200	12.08	11.98	17.80	13.01	11.73	15.81	17.44	15.78
900	5.99	11.94	8.68	12.81	5.75	15.72	8.25	15.54

Supplementary Table S.5: Sphere-based difference in SUV_{mean} between each AI-PET and Input-PET scan, respectively, and the respective ground truth Input-PET scan.

Mean and standard deviation of the absolute difference of SUV_{mean} values of all voxels within the sphere masks between each AI-PET and the respective ground truth Input-PET scan (column AI-PET) as well as between each Input- PET and the respective ground truth Input-PET scan (column Input-PET) for the different acquisition durations and for each of the four phantom setups (SBR4, thin: first column; SBR4, obese: second column; SBR10, thin: third column; SBR10, obese: fourth column). For each phantom setup, the Input-PET scan, which was considered as reference ground truth scan, is highlighted in gray. are in the same order of magnitude

The validation presented here using phantom data with a sphere-to-background ratio of 4 and thin patient setup yielded similar values for the absolute difference of SUV_{mean} values as the validation of the AI algorithm using clinical data when analyzing all lesions (0.9 ± 1.6) or a more specifically only lesions in lymph nodes (1.03 ± 1.00) in [Hosch]. A higher sphere-to-background contrast ratio of 10 in the phantom validation resulted in higher absolute SUV_{mean} differences than presented in [Hosch] for the validation of the AI algorithm using clinical data. Except for ultrashort scans with acquisition durations ≤ 20 s, the SUV_{mean} differences in all AI-PET scans of different acquisition durations were of a similar order of magnitude, which contradicts the statement in [Hosch] that the SUV differences are most likely a result of the ≥ 10-times reduced acquisition durations compared to ground truth scans. Rather, it indicates a structural problem of the applied GAN. In contrast, the acquisition times of the ground truth Input-PET scans can be shortened to an acquisition duration of approx. 1/10 of that of the ground truth scans without introducing SUV_{mean} differences compared to the ground truth scan. Even for Input PET scans with maximum short acquisition durations of 5 s, the SUV differences compared to ground truth scans were smaller than those of AI-generated AI-PET scans.

Acqusition duration (s)	SBR4, thin		SBR4, obese		SBR10, thin		SBR10, obese	
	Input-PET	AI-PET	Input-PET	AI-PET	Input-PET	AI-PET	Input-PET	AI-PET
**5**	0.38 ± 0.31	1.17 ± 0.7	0.48 ± 0.33	1.83 ± 1.16	0.16 ± 0.22	3.32 ± 1.38	0.47 ± 0.54	5.13 ± 1.47
**10**	0.33 ± 0.48	1.01 ± 0.67	0.26 ± 0.2	1.54 ± 1.11	0.14 ± 0.14	3.06 ± 1.32	0.27 ± 0.19	3.47 ± 1.6
**20**	0.16 ± 0.2	1.02 ± 0.71	0.19 ± 0.24	1.24 ± 1.03	0.11 ± 0.09	3 ± 1.44	0.38 ± 0.42	2.93 ± 1.33
**40**	0.08 ± 0.06	1.04 ± 0.78	0.14 ± 0.13	1.21 ± 1	0.14 ± 0.18	2.94 ± 1.43	0.17 ± 0.2	2.93 ± 1.59
**60**	0.09 ± 0.05	1.06 ± 0.84	0.07 ± 0.06	1.26 ± 1.04	0.12 ± 0.15	2.95 ± 1.48	0.09 ± 0.07	2.84 ± 1.51
**80**	0.03 ± 0.02	1.05 ± 0.82	0.13 ± 0.2	1.27 ± 0.96	0.09 ± 0.13	2.91 ± 1.46	0.1 ± 0.11	2.79 ± 1.49
**100**	0.04 ± 0.07	1.03 ± 0.81	0.11 ± 0.19	1.26 ± 0.95	0.07 ± 0.1	2.9 ± 1.45	0.1 ± 0.1	2.8 ± 1.52
**120**	0.03 ± 0.02	1.04 ± 0.83	0.07 ± 0.11	1.28 ± 0.97	0.02 ± 0.03	2.89 ± 1.44	0.08 ± 0.1	2.76 ± 1.5
**140**	0.02 ± 0.02	1.05 ± 0.83	0.05 ± 0.08	1.28 ± 1	0 ± 0	2.88 ± 1.44	0.07 ± 0.09	2.7 ± 1.42
**160**	0.02 ± 0.01	1.05 ± 0.83	0.02 ± 0.03	1.26 ± 0.99	0.04 ± 0.04	2.87 ± 1.44	0.07 ± 0.08	2.69 ± 1.43
**180**	0 ± 0	1.04 ± 0.83	0.02 ± 0.01	1.27 ± 1.01	0.03 ± 0.05	2.87 ± 1.44	0.02 ± 0.02	2.64 ± 1.38
**200**	0.02 ± 0.02	1.04 ± 0.82	0 ± 0	1.28 ± 1	0.03 ± 0.03	2.89 ± 1.45	0 ± 0	2.64 ± 1.39
**900**	0.05 ± 0.04	1.07 ± 0.86	0.1 ± 0.15	1.3 ± 1.08	0.07 ± 0.08	2.9 ± 1.46	0.07 ± 0.12	2.67 ± 1.46

Supplementary Table S.6: Sphere-based difference in SUV_{max} between each AI-PET and Input-PET scan, respectively, and the respective ground truth Input-PET scan.

Mean and standard deviation of the absolute difference of SUV_{max} values of all voxels within the sphere masks between each AI-PET and the respective ground truth Input-PET scan (column AI-PET) as well as between each Input- PET and the respective ground truth Input-PET scan (column Input-PET) for the different acquisition durations and for each of the four phantom setups (SBR4, thin: first column; SBR4, obese: second column; SBR10, thin: third column; SBR10, obese: fourth column). For each phantom setup, the Input-PET scan, which was considered as reference ground truth scan, is highlighted in gray.

The validation presented here using phantom data with a signal-to-background ratio of 4 and thin patient setup yielded similar values for the absolute difference of SUV_{max} values as the validation of the AI algorithm using clinical data when analyzing all lesions (1.5 ± 2.5) or a more specifically only lesions in lymph nodes (1.38 ± 1.73) in [Hosch]. An increase in the proportion of attenuation as in the SBR4-obese setup or a higher sphere-to-background contrast ratio of 10 in the phantom validation resulted in higher absolute SUV_{max} differences than presented in [Hosch] for the validation of the AI algorithm using clinical data.

Except for ultrashort scans with acquisition durations of 60 s and longer, the SUV_{max} differences in all AI-PET scans were of a similar order of magnitude, which contradicts the statement in [Hosch] that the SUV differences are most likely a result of the greatly reduced acquisition duration compared to ground truth scans. Rather, it indicates a structural problem of the applied GAN. In contrast, the acquisition duration of the ground truth Input-PET scans can be shortened to an acquisition duration of about half of that of the ground truth Input-PET scans without introducing strong SUV_{max} differences. Even for Input PET scans with acquisition durations of about 1/3 of that of the ground truth scans and longer, the SUV_{max} differences compared to ground truth scans were smaller than those of AI-generated AI-PET scans.

Acqusition duration (s)	SBR4, thin		SBR4, obese		SBR10, thin		SBR10, obese	
	Input-PET	AI-PET	Input-PET	AI-PET	Input-PET	AI-PET	Input-PET	AI-PET
**5**	9.75 ± 4.68	1.43 ± 1.57	17.46 ± 9.76	2.28 ± 1.82	11.23 ± 3.77	2.14 ± 2.42	20.92 ± 10.8	5.96 ± 2.19
**10**	5.69 ± 2.05	1.54 ± 1.53	9.1 ± 3.97	1.72 ± 1.86	8.37 ± 4.18	2.39 ± 1.93	11.38 ± 5.55	3.41 ± 2.61
**20**	3.06 ± 1.89	1.34 ± 1.35	4.96 ± 1.17	1.37 ± 1.6	4.08 ± 1.45	2.64 ± 2.21	9.2 ± 2.54	3.31 ± 1.28
**40**	1.58 ± 1.35	1.5 ± 1.11	3.53 ± 1.68	1.54 ± 1.55	2.16 ± 0.59	2.45 ± 2.32	4.01 ± 2.8	3.57 ± 2.03
**60**	0.74 ± 0.55	1.45 ± 1.15	2.35 ± 1.34	1.78 ± 1.55	1.49 ± 0.35	2.58 ± 2.38	2.39 ± 0.77	3.22 ± 2.09
**80**	0.59 ± 0.39	1.41 ± 1.15	1.69 ± 1.13	1.81 ± 1.34	1.07 ± 0.57	2.62 ± 2.28	1.07 ± 0.81	2.94 ± 2.25
**100**	0.48 ± 0.24	1.31 ± 1.13	1.2 ± 0.63	1.79 ± 1.24	0.5 ± 0.34	2.56 ± 2.18	0.68 ± 0.55	2.93 ± 2.38
**120**	0.38 ± 0.2	1.29 ± 1.16	0.79 ± 0.5	1.82 ± 1.37	0.21 ± 0.17	2.65 ± 2.17	0.75 ± 0.65	2.91 ± 2.27
**140**	0.22 ± 0.1	1.37 ± 1.12	0.64 ± 0.3	1.8 ± 1.49	0 ± 0	2.6 ± 2.22	0.66 ± 0.62	2.79 ± 2.08
**160**	0.1 ± 0.08	1.34 ± 1.12	0.35 ± 0.22	1.83 ± 1.47	0.22 ± 0.12	2.61 ± 2.17	0.46 ± 0.18	2.77 ± 2.17
**180**	0 ± 0	1.38 ± 1.1	0.19 ± 0.13	1.85 ± 1.58	0.3 ± 0.16	2.65 ± 2.17	0.21 ± 0.17	2.64 ± 2.07
**200**	0.12 ± 0.11	1.38 ± 1.09	0 ± 0	1.86 ± 1.56	0.3 ± 0.24	2.68 ± 2.2	0 ± 0	2.76 ± 2.07
**900**	0.52 ± 0.23	1.43 ± 1.22	0.72 ± 0.5	1.91 ± 1.71	0.86 ± 0.35	2.6 ± 2.34	1.43 ± 0.81	2.96 ± 2.23

Supplementary Table S.7: Image-to-image metric Peak Signal to Noise Ratio (PSNR) comparing Input-PET and AI-PET scans, respectively, with the respective ground truth scan.

Direct image-to-image comparison of each Input-PET (column Input-PET) and AI-PET scan (column AI-PET), respectively, with the respective Input-PET ground truth scan as of the Peak Signal to Noise Ratio (PSNR) analyzing SUV_{peak} of the largest sphere (37 mm diameter) and computed in decibel (dB) for the different acquisition durations and for each of the four phantom setups (SBR4, thin: first column; SBR4, obese: second column; SBR10, thin: third column; SBR10, obese: fourth column). For each phantom setup, the Input-PET scan, which was considered as reference ground truth scan, is highlighted in gray. PSNR was calculated using a mask for the entire phantom. For each phantom setup, the PSNR in all AI-PET data reached the value of the respective ground truth scan, which means that the ratio between the maximum possible signal power and the power of the distorting noise was approximately that of the ground truth scan and thus the quality of the PET image was similarly good. An increase in the acquisition time of the Input-PET scan compared to the length of the ground truth Input-PET scan led to a strong increase in PSNR due to a reduction in image noise with increasing image statistics, thus illustrating the improvement in image quality. However, an application of the AI algorithm to Input-PET data with higher acquisition duration than the ground truth data (here 900 s) resulting in a strong reduction of PSNR in AI-PET scans compared to ground truth scans, meaning a deterioration of image quality. The PSNR is an absolute numerical value that depends not only on the noise, but also on the absolute SUV in the image. Since the SUV values can differ strongly between clinical data and phantom data, e.g. due to high uptake in the bladder in clinical data and different signal-to-background contrast ratios in phantom data, absolute PSNR values are not comparable between clinical and phantom data.

Acqusition duration (s)	SBR4, thin		SBR4, obese		SBR10, thin		SBR10, obese	
	Input-PET	AI-PET	Input-PET	AI-PET	Input-PET	AI-PET	Input-PET	AI-PET
**5**	5.29	31.89	4.02	17.48	13.3	56.13	7.3	35.34
**10**	8.11	35.4	5.43	26.55	17.77	58.35	12.91	57.26
**20**	11.74	36.47	7.47	30.61	26.11	59.53	17.09	63.85
**40**	15.55	36.71	10.83	35.33	37.34	61.22	26.03	69.14
**60**	18.84	36.17	13.45	35.86	44.46	61.96	31.39	71.64
**80**	22.15	34.79	15.68	36.64	52.03	62.42	35.05	70.95
**100**	25.17	35.46	17.4	37.68	58.41	62.46	39.77	67.71
**120**	28.19	35.55	19.31	37.51	64.41	62.53	44.3	70.02
**140**	30.13	35.6	20.27	37.79	69.07	62.65	47.89	70.29
**160**	31.98	35.58	22.57	37.88	73.71	62.78	51.87	71.51
**180**	33.53	35.9	23.79	38.02	78.81	62.54	54.35	71.7
**200**	34.03	35.8	24.33	38.21	82.16	62.69	56.73	68.83
**900**	71.6	36.7	48.63	37.58	163.58	62.28	116.55	70.47

Supplementary Table S.8: Image-to-image metric Mean Absolute Error (MAE) comparing each Input-PET and AI-PET scans, respectively, with the respective ground truth scan.

Direct SUV-based voxelwise comparison of each Input-PET (column Input-PET) and AI-PET scan (column AI-PET), respectively, with the respective Input-PET ground truth scan as of the Mean Absolute Error (MAE) for the different acquisition durations and for each of the four phantom setups (SBR4, thin: first column; SBR4, obese: second column; SBR10, thin: third column; SBR10, obese: fourth column). For each phantom setup, the Input-PET scan, which was considered as reference ground truth scan, is highlighted in gray. MAE was calculated using a mask for the entire phantom.

For very low acquisition durations ≤ 20 s, the AI-generated AI-PET scans featured lower MAE values compared to the Input-PET scans of the same acquisition durations, meaning that the AI was able to reduce the SUV differences in ultra-short PET scans. However, especially due to the stripe artifacts in the AI-PET scans, which are caused by the 2.5D GAN structure of the AI network, absolute SUV differences between AI-PET and ground truth scan are quite high, resulting in averagely high MAEs ranging between 0.232 and 0.333 in the AI-PET scans. For the same reason, MAE was higher all AI-PETs compared to the Input-PET scans with the same acquisition durations for acquisition durations ≥ 80 s, which suggests a deterioration in quantitative accuracy.

The MAE is an absolute numerical value that depends on the absolute SUV in the image. Since the SUV values can differ strongly between clinical data and phantom data, e.g. due to high uptake in the bladder in clinical data and different signal-to-background contrast ratios in phantom data, absolute MAE values are not comparable between clinical and phantom data.

**Acqusition duration (s)**	**SBR4, thin**		**SBR4, obese**		**SBR10, thin**		**SBR10, obese**	
	**Input-PET**	**AI-PET**	**Input-PET**	**AI-PET**	**Input-PET**	**AI-PET**	**Input-PET**	**AI-PET**
**5**	0.731	0.238	0.903	0.333	0.644	0.235	0.85	0.282
**10**	0.534	0.235	0.703	0.262	0.462	0.232	0.626	0.251
**20**	0.376	0.244	0.503	0.246	0.319	0.233	0.445	0.238
**40**	0.251	0.253	0.342	0.244	0.208	0.236	0.301	0.236
**60**	0.191	0.257	0.264	0.246	0.153	0.237	0.232	0.237
**80**	0.151	0.259	0.213	0.247	0.115	0.237	0.186	0.237
**100**	0.121	0.259	0.174	0.247	0.084	0.237	0.153	0.238
**120**	0.096	0.26	0.142	0.248	0.054	0.237	0.124	0.238
**140**	0.073	0.26	0.114	0.248	0	0.237	0.1	0.238
**160**	0.048	0.26	0.087	0.248	0.047	0.238	0.076	0.238
**180**	0	0.26	0.058	0.248	0.063	0.238	0.051	0.238
**200**	0.043	0.26	0	0.248	0.073	0.238	0	0.238
**900**	0.122	0.264	0.155	0.253	0.122	0.241	0.135	0.241
